# Therapeutic Importance of Kaempferol in the Treatment of Cancer through the Modulation of Cell Signalling Pathways

**DOI:** 10.3390/molecules27248864

**Published:** 2022-12-13

**Authors:** Malak Yahia Qattan, Mohammad Idreesh Khan, Shudayyed Hasham Alharbi, Amit Kumar Verma, Fatimah A. Al-Saeed, Alduwish Manal Abduallah, Azza A. Al Areefy

**Affiliations:** 1 Department of Health Sciences, College of Applied Studies and Community Service, King Saud University, KSA- 4545, Riyadh 11451, Saudi Arabia; 2 Department of Clinical Nutrition, College of Applied Health Sciences in Ar Rass, Qassim University, Ar Rass 51921, Saudi Arabia; 3 Pharmacy Department, Maternity and Children Hospital (MCH), Qassim Cluster, Ministry of Health, Buraydah 52384, Saudi Arabia; 4 Department of Pharmacology and Toxicology, College of Pharmacy, Qassim University, Buraydah 51452, Saudi Arabia; 5 Department of Biotechnology, Jamia Millia Islamia University, New Delhi 110025, India; 6 Department of Biology, College of Science, King Khalid University, Abha 61413, Saudi Arabia; 7 Research Centre for Advanced Materials Science (RCAMS), King Khalid University, P.O. Box 9004, Abha 61413, Saudi Arabia; 8 Department of Biology, College of Science and Humanities in Al-Kharj, Prince Sattam Bin Abdulaziz University, Alkarj 11942, Saudi Arabia; 9 Department of Clinical Nutrition, College of Applied Medical Sciences, Jazan University, Jazan 45142, Saudi Arabia; 10 Nutrition & Food Science Department, Faculty of Home Economics, Helwan University, P.O. Box 11795, Cairo 11281, Egypt

**Keywords:** kaempferol, flavonoids, cell signalling pathways, anti-inflammatory, anti-cancer activity

## Abstract

Plant-derived flavonoids are considered natural nontoxic chemo-preventers and have been widely studied for cancer treatment in recent decades. Mostly all flavonoid compounds show significant anti-inflammatory, anticancer and antioxidant properties. Kaempferol (Kmp) is a well-studied compound and exhibits remarkable anticancer and antioxidant potential. Kmp can regulate various cancer-related processes and activities such as cell cycle, oxidative stress, apoptosis, proliferation, metastasis, and angiogenesis. The anti-cancer properties of Kmp primarily occur via modulation of apoptosis, MAPK/ERK1/2, P13K/Akt/mTOR, vascular endothelial growth factor (VEGF) signalling pathways. The anti-cancer property of Kmp has been recognized in several in-vivo and in-vitro studies which also includes numerous cell lines and animal models. This flavonoid possesses toxic activities against only cancer cells and have restricted toxicity on healthy cells. In this review, we present extensive research investigations about the therapeutic potential of Kmp in the management of different types of cancers. The anti-cancer properties of Kmp are discussed by concentration on its capability to target molecular-signalling pathway such as VEGF, STAT, p53, NF-κB and PI3K-AKT signalling pathways. The anti-cancer property of Kmf has gained a lot of attention, but the accurate action mechanism remains unclear. However, this natural compound has a great pharmacological capability and is now considered to be an alternative cancer treatment.

## 1. Introduction

The cancer prevalence and incidence of harmful disorders is increasing among developing and developed nations [1]. GLOBOCAN 2020 data reported 19.3 million new cancer cases and around 10 million cancer related deaths across the world [2]. Though the advancement in treatment and diagnostic methods and cancer-awareness programmes have caused incredible decrease in mortality of cancer in United States, but the prevalence of cancer is yet increasing unceasingly [1]. Intake of unhealthy diet and alcohol, stress, physical inactivity and smoking are the key factors responsible for cancer prevalence in under developed, developing and developed nations [3]. Chemo- and radio- therapy, targeted treatment, surgery, and biological therapy are widely used therapies used in the treatment of cancer from decades. KRAS inhibitors such as cetuximab in colorectal carcinoma, inhibitors of epidermal growth factor receptor such as fefitinib, osimertinib, afatinib and erlotinib in non-small-cell lung carcinoma (NSCLC), BCR-ABL tyrosine kinase inhibitors such as Imatinib mesylate in leukaemia, BRAF inhibitors such as encorafenib, dabrafenib and vemurafenib in melanomas, herceptin and tamoxifen in breast cancers have been extensively utilised in treatment of different cancer types [4]. Cancerous cells can escape mortality by achieving resistance to several therapeutic methods, and this can attenuate the projected outcomes of the cancer treatments [5,6,7]. Nowadays actively goes search of new natural compounds with anticancer potential as well. New and alternate therapeutic methods have been required to treat patients of cancer. Several in-vitro research in combination of ex-vivo research have demonstrated the anticancer impacts of natural substances like flavonoids [8,9,10,11]. In a study it was found that dietary intakes of flavonols, including kaempferol, quercetin, myricetin, and isorhamnetin may be associated with reduced risk of developing Alzheimer dementia [12]. Annona purpurea contains five acetogenins named annopurpuricins A–E, which are active against tumoural cell lines in a subnanomolar range. Fagopyrin is a naphthodianthrone found in Buckwheat (*Fagopyrum esculentum* L.) have antioxidant and anticancerogenic activity. 

One of the major component in flavonoids among plants are quercetin (Qu), kaempferol (Kmp) [13,14] which have anti-oxidant and anti-inflammatory property [15]. Quercetin and kaempferol are widely distributed in fruit and vegetables. High concentrations of quercetin are found in a few foods such as onion, asparagus, and berries, and small quantities are found in many different fruit and vegetables. The richest plant sources of kaempferol (mg/100 g fresh weight) are green leafy vegetables, including spinach and kale, and herbs such as dill, chives, and tarragon [16]. Kmp has been overlooked by the researchers because of a very few in-vitro researches have evaluated the potential of Kmp in treatment of cancers. In very recent times, few studies reported that antioxidant property of flavonoids offered novel therapeutic approaches for chemotherapy in treatment of cancer. Some studies described that Kmp-stimulate activation of antioxidants that might play a vital function in NSCLC-H460 cancerous cell apoptosis [17]. Numerous researchers have proven the antioxidant, anticarcinogenic, antidiabetic, cardioprotective, neuroprotective and antimicrobial properties of kaempferol and its glycosides [18]. Additionally, several research stated that Kmp considerably prevents the cancerous growth in in-vitro conditions which leads to apoptosis in cancerous cells [19,20,21].

Many cancer-related molecules like matrix metallopeptidases (MMPs), proapoptotic and anti-apoptotic proteins, various growth factors, cyclins, and cyclin-dependent kinases (CDKs) have been demonstrated to be regulated by Kmp [22,23,24,25,26,27]. Moreover, Kmp also exhibits synergetic effect where it enhances the anti-tumor activities of several anticancer drugs. 

**Figure 1:**
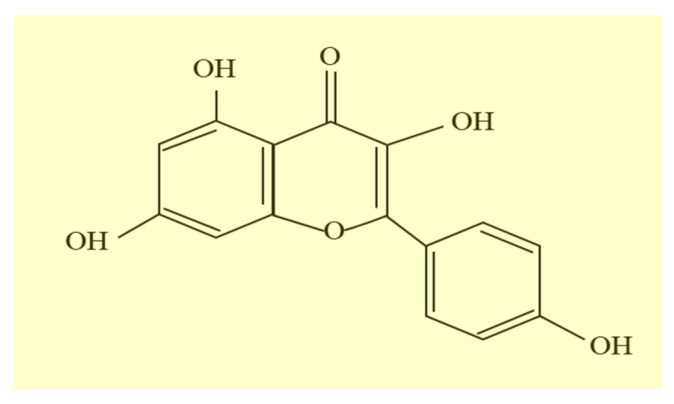
Chemical structure of Kaempferol [26].

Kmp is (3,4′,5,7-tetrahydroxyflavone) is a yellowish flavonoid compound which has 4 hydroxy-groups at -3, -4′, -5 and -7 positions (Figure 1) [28]. It can be present in several parts of plants like leaves, flowers, seeds, vegetables, and fruits [25,27,29]. Kmp and its glycosides have antidiabetic, anticancer, neuro protective, antioxidant, antitumor, anti-inflammatory, anti-microbial and cardio protective properties [30]. Epidemiologic researches exhibited that greater consumption of Kmp is linked with reduced occurrence of various cancers including colorectal, hepatic, pancreatic, urinary bladder, ovarian, and gastric cancers [28,29]. Due to several anticancer activities, intake of Kmp and associated uses are getting vast interest among researchers in the cancer treatment [31,32,33]. Inhibition of cancerous cells growth is generally attained by preventing cancerous cells proliferation via enhancing apoptosis [34,35,36]. Certainly, Kmp prevents growth of cancerous cells by initiating G2-M stage cell-cycle arrest, apoptosis, down-regulation of signalling pathways such as phosphatidylinositol 3-kinase-protein kinase B (PI3K-PKB), epithelial mesenchymal transition (EMT) markers’ expression such as SNAI1, E-cadherin and N-cadherin and (MMP2) markers [37,38]. Kmp also stimulates the activation of caspases such as caspase-9, caspase-7, caspase-3 and poly (ADP-ribose) polymerase (PARP) proteins which involve in initiation and execution of apoptosis [39], hence, inhibiting the reactive oxygen species (ROS) accumulation included in the development of cancer [40]. It has been also stated that Kmp can maintain normal viability of cells and prevent angiogenesis [40].

**Figure 2:**
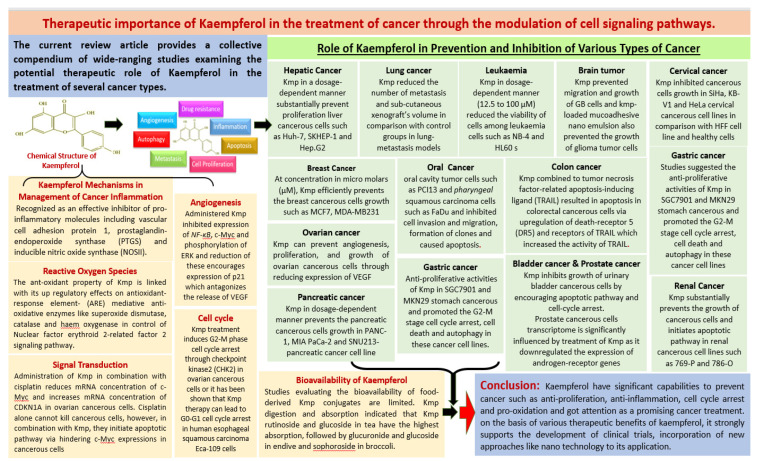
Graphical representation of literature review.

Due to effective role in cancer inhibition, extensive pharmacological properties and numerous health promoting benefits of Kmp, this current review article provides a collective compendium of wide-ranging studies examining the potential therapeutic role of Kmp in treatment of several cancer types. Since Kmp asserted to carry the anti-inflammatory, antioxidant and anti-tumor properties and capability to prevent the cancerous cells proliferation, it has been widely studied as a chemo preventive agent in numerous cancerous models and Kmp modulates several cell signalling molecules. Figure 2 graphical representation of literature review.

## 2. Major Mechanisms of Kaempferol (Kmp) in Management of Cancer 

### 2.1. Inflammation 

Inflammation is a biologically complex protective body’s reaction rising due to dangerous stimuli and damaged cells. Many diseases are characterized by inflammation including allergy, transplant rejection, preperfusion injury, hepatitis, glomerulonephritis, asthma, autoimmune disorders, celiac disease, intestinal inflammation and cancer [41]. Hence, inflammation is a biologically, self-protecting body’s reaction in times of problem which eliminates injured and damaged cells and starts the healing process [42]. It has been suggested that chronic inflammation is linked with the progression of several disorders such as neurodegeneration, cancer, and arthritis [43,44,45]. Kmp has been recognised as an effective inhibitor of pro-inflammatory molecules including vascular cell adhesion protein 1, prostaglandin-endoperoxide synthase (PTGS) and inducible nitric oxide synthase (NOSII) [46,47]. Anti-inflammatory effects of Kmp are mostly facilitated by downregulation of numerous sequence-specific DNA-binding factors like STAT, nuclear factor kappa-light-chain-enhancer of activated B cells (NF-κB), which have the capability to encourage the pro-inflammatory cytokines activation [48]. A study analysed the anti-inflammatory property of Kmp in hepatic cell lines and found that Kmp reduced the PTGS, NOSII, C-reactive protein (CRP) expression by altering NF-κB signalling pathway [49]. The ability of Kmp to deal with inflammation is one of its critical and considerable features in cancer prevention (Table 1). When lipopolysaccharide-induced macrophages treated with Kmp, it resulted into downregulation of PTGS, NOSII and tumor necrosis factor-alpha (TNF-alpha) at translational- and transcriptional- levels through inhibiting sequence-specific DNA-binding factors such as Activator protein 1 (AP1) and NF-κB [50,51]. Furthermore, protein-kinase signalling cascades mechanism directed by interleukin-1 receptor-associated kinase (IRAK)-1, -4, Syk and Src which are generally take part in AP1 and NF-κB factors activation and could prevented by Kmp [52]. In diseases like Crohn’s disease or rheumatoid arthritis, uncontrolled inflammation can cause immune system arrest where immune system harms normal healthy cells. Chronic inflammation is associated with a susceptibility in development of cancer [53]. Stomach ulcer is linked with an increasing risk of peptic cancer and mesothelioma can be tracked back to irritation caused by asbestos. It has been reported that flavonoid (particularly Kmp) rich diet is correlated with decreased level of serum interleukin-6 which is an inflammatory cytokine [54]. In aldosterone induced human umbilical-vein endothelial cell (HUVEC), Kmp has been reported to downregulate the expression of ROS-dependent cytokines such as osteopontin which activates and stimulates NF-κB and p38-mitogen-activated protein kinases (p38-MAPK) signalling [47]. Hence, studies are recommending Kmp as a promising anti-inflammatory drug and it can be proposed for in-vivo trials.

### 2.2. Reactive Oxygen Species (ROS)

Metabolic pathways generate ROS in the body which are key resource of destructive oxidative stress [55,56]. Though humans have antioxidant enzymes as defence mechanisms which continuously neutralises ROS, but high ROS concentration causes infections, senescence, cerebrovascular accident, autoimmune disorders, cardiovascular arteriosclerosis, oxygen poisoning, Parkinson’s disorder and becomes lethal [55,57]. Studies suggested that flavonoids can be efficient secondary-metabolites against oxidative stress-related diseases [58]. Kmp increases the anti-oxidant enzymes expressions at high concertation and at low-concentration it scavenges hydroxyl (OH) radical and peroxonitrite radical [30]. The antioxidant property of Kmp is linked with its up regulatory effects on antioxidant-response element- (ARE) meditative anti-oxidative enzymes like superoxide dismutase, catalase and haem oxygenase in control of Nuclear factor erythroid 2-related factor 2 signalling pathway [59]. Kmp can be used in prevention of susceptibility of oxidation of low density lipoproteins (LDL) and aggregation of platelets [60]. Both Wahab et al. 2014 and Choe et al. 2012 studied antioxidant property of Kmp by extracting and purifying Kmp from Senna alata beans and Rhodiola sachalinensis roots respectively [61,62]. It has been observed that Kmp reduced the thiobarbituric-acid reactive substances and red blood corpuscles lysates and upregulated the level of enzymatic antioxidants such as superoxide dismutase, glutathione perxidases (GSHPx) and catalase when 1,2-dimethylehydrazine (DMH)-induced-colon cancer male Wistar-rats treated with Kmp [63]. Similarly, researchers studied hepatoprotective effects of Kmp by increasing in carbon tetrachloride (CCl_4_)-induced liver damage in rodents [64,65]. Kmp reduces the level of reactive oxygen and increases the survival of cell in oxidatively stressed HT 22 neuronal cells and reduces oxidative DNA-damage in isolated human lymphocytes [66].

### 2.3. Angiogenesis

**Figure 3:**
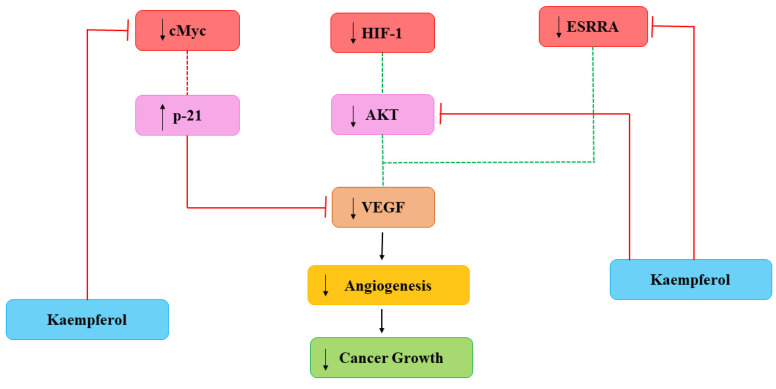
Kaempferol effects on Angiogenesis. HIF-1: hypoxia inducible factor-1; VEGF: vascular endothelial growth factor; ESRRA: estrogen related receptor alpha. Dotted lines signify earlier processes that have decreased due to Kmp [23].

Cancerous cells also need nutrients and oxygen to survive provided with networks of capillaries. Angiogenesis is linked with repair of damaged cells and reproductive development via formation of new capillaries which is mediated by growth molecules, endostatins, adhesion molecules etc. [67]. Main mediator in angiogenesis is VEGF and formation of new capillaries aimed to meet increasing requirements of the tumor [68]. Current studies have demonstrated the efficiency of Kmp in reducing angiogenesis of cancer in in-vitro and in-vivo by preventing secretion of VEGF in human cancerous cells [69,70]. A study reported that Kmp prevented VEGF secretion in MDA-MB-231 cancerous cells and decreased the concentration of VEGF-mRNA among ovarian cancerous cell lines [71]. Level of VEGF proteins was significantly influenced by Kmp, indicating action-mechanism involved in translation [67]. Kmp inhibits angiogenesis and expression of VEGF via ERK-NFkappaB-cMyc-p21 pathways [70]. Administered Kmp inhibited expression of NF-κB, c-Myc and phosphorylation of ERK and reduction of these encourages expression of p21 which antagonizes the release of VEGF [68]. Moreover, Kmp also affected regulators of VEGF. Kmp reduces the level of hypoxia inducible factor (HIF)-1 and inhibits phosphorylation of AKT signalling pathway and it blocks signalling mechanisms which involves in enhanced VEGF secretion [67]. Kmp also inhibits activity of estrogen related receptor alpha (ESRRA) by reducing its mRNA level. ESRRA is linked with oestrogen-activity and considered as a cancer promoter. Kmp is an opponent of VEGF and attacks production of VEGF from every path (Figure 3) [23].

### 2.4. Signal Transduction

Numerous interleukin-6 related signalling pathways have been linked and found with increased migration, invasion, and proliferation of several tumor cells. Interleukin-6 binds with interleukin-6 specific binding receptor-α and activates the dimerization of signal-transducer receptor called glycoprotein 130 and causes its phosphorylation, followed by Janus tyrosin kinase (JAK) activation [72]. These incidents cause the activation of several signal-transduction pathways, like signal-transducer and activator of transcription (STAT), PI-3 kinase signalling pathways [72]. In all these pathways, STAT3 signalling pathway is the mostly analysed/studied cytokine-signalling pathway [73,74]. STAT3 is a member of STAT-family of transcription-factors and plays an important role in cancer related inflammation. STAT3 is often de-regulated in several kinds of cancer and function as an onco-gene in tumorigenesis [75]. STAT3 activation causes expression of down-stream genes which regulate main cell responses (includes survival of cancerous cell, cell invasion and proliferation) like BCL2, cyclin-D1 and MMP-2 [76]. STAT3 plays important role in tumorigenesis and in progression of cancer which allow STAT3 to arise as a promising molecule target in the treatment of cancer. Basu et al. (2020) observed that at high concentration, Kmp prevented interleukin-6 induced-phosphorylation of STAT3 [77]. A study conducted by Yang et al. (2019) concluded that Kmp inhibits STAT3 signalling pathway [78]. 

Phosphatidylinositide-3-Kinase (PI3K) is an important signal-transducing enzyme which regulates cell differentiation, survival, angiogenesis, proliferation, and apoptosis [79,80]. It is vital for AKT activation and has an important role in pathological as well as physiological signalling processes. Due to the repeated activation of PI3K-AKT mechanism in cancer, it is a key drug target [81,82,83,84,85]. PI3K is a lipid-kinase which causes phosphorylation PIP2–PIP3 and it is the PDK and AKT activation site. Family of PI3K has three different classes viz, class I, II and III and these classes are different in distribution of tissue, in function, preference of substrate, activation pathway and structure [86,87,88]. PI3K-dependent AKT activation results into multi step method which involve both phosphorylation as well as translocation [89]. Activation of AKT includes the phosphorylation of two residues: serine 473 (Ser473) at carboxy-terminal and threonine 308 (Thr308) on activation loop. Ser473 is phosphorylated by PDK2 while PDK1 phosphorylated by Thr308 [90,91]. PDK1 is an important kinase needed for normal development in mammals [92]. AKT has three isoforms: AKT-1, AKT-2 and AKT-3 based on their different biological activities and distribution of tissue. AKT-1 plays a vital role in angiogenesis and cell survival regulation [86,93,94]. PI3K activation is counter-production to apoptotic pathway and due to this, several drugs related to cancer treatment concentrate on inhibition of this pathway. Chin et al. (2018) reported that Kmp in dosage-dependent manner significantly reduced the mTOR and AKT phosphorylation and level of PI3K protein [95]. Another study reported that Kmp repressed the growth of colorectal cancerous cells by preventing the activation of PI3K-AKT signalling pathways [96].

**Figure 4:**
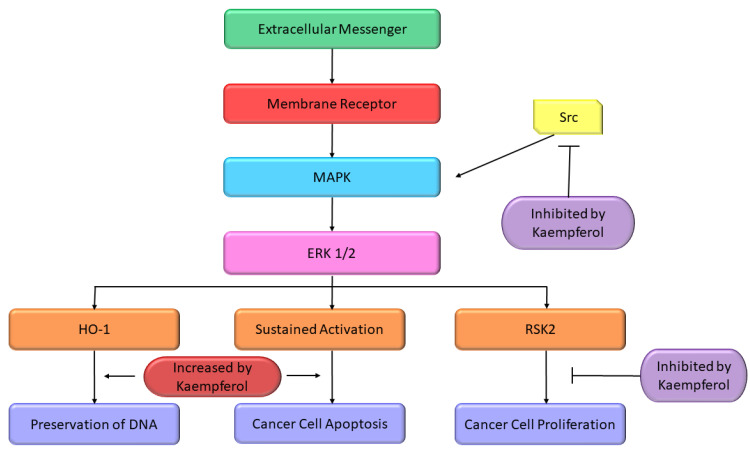
Effect of Kmp on MAPK pathway [26].

Some studies reported the apoptosis inducing properties of Kmp which can be partly accredited to its impacts on pathway of MAPK. In A-549 and MCF-7 cell lines, initiation of MAPK pathway is a key factor in Kmp-induced apoptosis. Moreover, Kmp-mediated activation of MAPK can block DNA damage which leads to transformation of cell. Kmp presence increases the expression of haemoxygenase-1 gene (HO-1), which triggers the rise in antioxidant ability of cells [97]. Treatment of Kmp significantly increased the viability of cells in response to oxidative stress, which involves unstable free radicals susceptible to damage DNA. Thus, Kmp-induced MAPK induction defends healthy cells from converting into cancer cells. RSK2 is a major suppressor of apoptosis, it downregulates the BAD, a protein which promotes apoptosis and upregulates the Bcl-2 level [98]. It has been observed that Kmp directly binds to RSK2 protein particularly at lysine-100 (Lys) and valine-82 (Val) positions, which plays an important role in RSK2 functioning [99]. Thus, Kmp paralyzes the RSK2. Obviously, treatment dropped Bcl level and increase concentration of tumor suppressor protein such as p53 and BAD [98]. Moreover, Kmp has also been reported to interrupt activity of Src-kinase [100]. MAPK is activated by Src in pro growth situation, which activates the COX-2 protein, and occurrence of COX-2 is a cautionary marker for skin tumor [101]. MAPK-ERK pathway is modified at various crucial sites by Kmp (Figure 4).

Hence, Kmp affects STAT3, PI3K signalling and MAPK pathway and exhibits significant potential in manipulation of cell-signalling pathways in apoptosis initiation and leaves normal cells alone.

### 2.5. Cell Cycle

A cell cycle is repeating series of events which involves copying of contents of cell and following division. Cells are continuously subject to DNA mutation that is harmful for cells but hardly results in cells production which can avoid the normal restrictions and flourish as pathologic tumors [102]. The development and progression of cancer is often associated with disruption or dysregulation of normal cell-cycle progression. Cells react to damage in DNA by stopping cell cycle progress and / or by enduring apoptosis [102].

**Figure 5:**
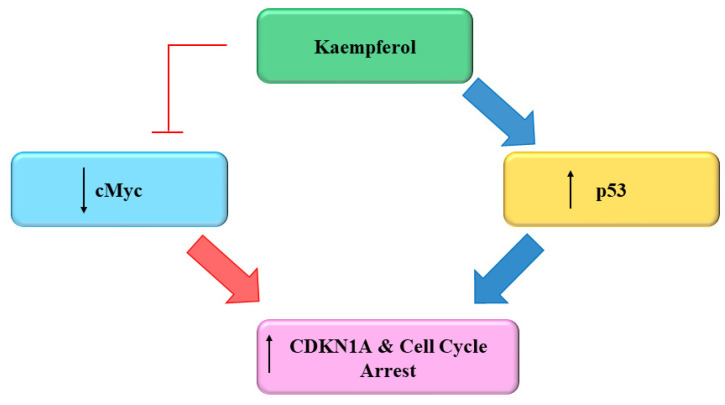
Effect of Kaempferol on the Cell Cycle [112].

Several flavonoids and natural chemo preventers including Kmp have been observed to precisely regulate numerous proteins which are involved in cellular homeostasis and cell cycle, whose de-regulation may play a role in carcinogenesis [103,104]. The ability of Kmp to induce cell cycle arrest have been observed in several cell cycles like in a study conducted by Gao et al. (2018) found that Kmp treatment induces G2-M phase cell cycle arrest through checkpoint kinase2 (CHK2) in ovarian cancerous cells [105] and Xu et al. (2008) in their study observed that Kmp induces G2-M phase cell cycle arrest in cervical cancerous cells [106], it has been reported that Kmp therapy can lead to G0-G1 cell cycle arrest in human esophageal squamous carcinoma Eca-109 cells [107]. Kmp treatment increased the level of p53 in MDA-MB-453 breast cancerous cells [108]. Furthermore, gene c-Myc is usually overexpressed in cancerous cells which leads to uncontrolled cell proliferation [109]. Studies showed that enhanced c-Myc level antagonized mRNA concentration of CDKN1A [110], administration of Kmp in combination with cisplatin reduces mRNA concentration of c-Myc and increases mRNA concentration of CDKN1A in ovarian cancerous cells. Cisplatin alone cannot kill cancerous cells, however, in combination with Kmp, they initiate apoptotic pathway via hindering c-Myc expressions in cancerous cells [111]. p53 is famous tumor suppressor protein generally indicated as ‘guardian of genome’ [111]. Repairing of damaged DNA is generally regulated by p53 [111]. Luo et al. (2011) observed that Kmp prevented phosphorylation of AKT signalling but upregulated the p53 expression and induced apoptosis in ovarian cancerous cells (Figure 5) [98]. Kmp is a useful flavonoid with genuine ability in disrupting growth of cancer and deserves more study into its impact on the cell cycle. A versatile chemoprophylactic molecule, kmp appears to play a role in each part of growth of cancer. Indeed, there persist a host of kmp-sensitive genes awaiting to be studied [112]. Kmp can efficiently prevent the proliferation and activation of mice T-lymphocytes in response to ConA, and can arrest cell cyle at G2/M and S phases [113].

### 2.6. Remodeling Tumor Metabolism

Metabolic remodeling is a phenomenon of the occurrence and development of tumors. It provides energy and material to the cells for survival and proliferation and prepares cells to survive in the harsh microenvironment [114]. Kaempferol inhibit both growth and migration of glioma cells, even when kaempferol was loaded to mucoadhesive nanoemulsion (KPF-MNE) or kaempferol-loaded nanoemulsion (KPF-NE) [27].

**Table 1:** Major mechanism of action of Kaempferol (Kmp) in cancer management.

Major Mechanism	Outcome of the Study	Refs
Inflammation	Kmp has been recognised as an effective inhibitor of pro-inflammatory molecules including vascular cell adhesion protein 1, prostaglandin-endoperoxide synthase (PTGS) and inducible nitric oxide synthase (NOSII)	[46,47]
Inflammation	Anti-inflammatory effects of Kmp are mostly facilitated by downregulation of numerous sequence-specific DNA-binding factors like STAT, nuclear factor kappa-light-chain-enhancer of activated B cells (NF-κB) which have the capability to encourage the pro-inflammatory cytokines activation	[48]
Reactive Oxygen Species (ROS)	The ant-oxidant property of Kmp is linked with its up regulatory effects on antioxidant-response element- (ARE) mediative anti-oxidative enzymes like superoxide dismutase, catalase, and haem oxygenase in control of nuclear factor erythroid 2-related factor 2 signalling pathway	[59]
Reactive Oxygen Species (ROS)	Kmp reduced the thiobarbituric-acid reactive substances and red blood corpuscles lysates and upregulated the level of enzymatic antioxidants such as superoxide dismutase, glutathione perxidases (GSHPx) and catalase when 1,2-dimethylehydrazine (DMH)-induced-colon cancer male Wistar-rats treated with Kmp	[63]
Angiogenesis	Kmp prevented VEGF secretion in MDA-MB-231 cancerous cells and decreased the concentration of VEGF-mRNA among ovarian cancerous cell lines	[71]
Angiogenesis	Administered Kmp inhibited expression of NF-κB, c-Myc and phosphorylation of ERK and reduction of these encourages expression of p21 which antagonizes the release of VEGF	[70,71]
Signal transducer and activator of transcription 3 (STAT3)	At high concentration, Kmp prevented interleukin-6 induced-phosphorylation of STAT3	[77]
Phosphatidylinositide-3-kinases (PI3K)-AKT pathways (PI3K-AKT)	Kmp repressed the growth of colorectal cancerous cells by preventing the activation of PI3K-AKT signalling pathways	[96]
Cell cycle	Kmp treatment induces G2-M phase cell cycle arrest through checkpoint kinase2 (CHK2) in ovarian cancerous cells or it has been shown that Kmp therapy can lead to G0-G1 cell cycle arrest in human esophageal squamous carcinoma Eca-109 cells	[105,106,107]
Cell cycle	Administration of Kmp in combination with cisplatin reduces mRNA concentration of c-Myc and increases mRNA concentration of CDKN1A in ovarian cancerous cells	[111]

## 3. Role of Kaempferol in Prevention and Inhibition of Various Types of Cancer

### 3.1. Hepatic Cancer

Hepatocellular carcinoma (HCC) is highly encountered hepatic cancer in adults [115]. It has been reported that Kmp in a dosage-dependent manner substantially prevent proliferation of liver cancerous cells such as Huh-7, SKHEP-1 and Hep.G2. Kmp plays an important role in inhibition and prevention of cancer through modulating various biological activities (Table 2). Additionally, 2-acetylaminofluorene and N-Nitrosodiethylamine-stimulated hepatocellular carcinoma from mice treated with combination of Kmp and luteolin prevented cancerous cells growth and caused apoptosis [116,117]. Kmp initiates apoptosis and triggers G2-M stage cell cycle arrest, hence, inhibiting invasion and migration of cancerous cells. Kmp can discharge cytochrome-c by generating ROS stimulates initiation of mitochondria swelling and loss of MtMP and increase the caspase 3 levels [115,116,117]. Kmp also increases the expression of non-receptor tyrosine-protein kinase (TYK-2), Janus kinase-1 (JAK-1), microtubule associated protein-1A-1B light chain-3 (MAPILC3), STAT1-2, autophagy related genes -5, -7 and -12, beclin-1 and phosphatase and tensin homolog (PTEN) and reduced the expression of cytokine signalling-3 (SOCS-3), PI3K-AKT-mTOR, miRNA-21, signal transducer and activator of transcription-3 (STAT-3), phosphorylated-mTOR signalling pathways and HIF1 in HCC [116,117,118,119]. 

### 3.2. Lung Cancer

Lung cancer is one of the most diagnosed cancers globally with an average survival rate of 5 years in most of the countries [120]. Lung cancer mainly is of two types- adenocarcinomas and NSCLC [121]. Harmful diet such as high intake of salt, low intake of fruits and vegetables, exposure to chemical carcinogens and smoking tobacco are the main risk factors linked with lung cancer [122]. Several flavonoids role in lung cancer have been examined by researchers [123,124] and in a cell line based study, it has been reported that Kmp inhibited the NSCLC, A549 cancerous cells [125,126,127,128], reduced formation of colonies and caused apoptosis [129]. Kmp significantly inhibited the migration of cells, suppressed epithelial mesenchymal transition and regained E-cadherin loss [130]. Kmp upregulated the expression level of Fas, transcription of miRNA-340, caspase 3, 7, 8, 9 and Bax and down-regulated the expressions of PI3K-AKT, Extracellular signal-regulated kinase signalling pathways, Mitogen-activated protein kinase (MEK)-1/2, MMP-2, B-cell lymphoma-extra-large and Bcl 2 which are involved in apoptotic pathways [17,125,126,127,128,129]. A study showed that Kmp reduced the number of metastasis and sub-cutaneous xenograft’s volume in comparison with control groups in lung-metastasis models [129].

### 3.3. Prostate Cancer

Prostate cancer (PCa) is among the male population is prominent cause of death worldwide and there is a requirement of efficient therapy for this disorder [131]. Kmp in dose-dependent manner prevents the proliferation of PCa cells [131], through up-regulation of expression levels of PARP and caspase-3, -9, -8 proteins [131,132]. Colony stimulating factor-2 activates the immune system of host and enable the immune surveillance of host through dendritic cells (DCs), hence, indicating a potential therapeutic in PCa treatment [132]. It has been observed that Kmp induces colony stimulation factor-2 release in PC3 cancerous cells and increases the DCs chemotaxis by activating protein kinase C, phosphor lipase C and MEK-1/2 [129]. Apparently, the PCa cells transcriptome is significantly influenced by treatment of Kmp as it downregulated the expression of androgen-receptor genes [133]. While orally consumed Kmp in mice did not demonstrate substantial toxicity and considerably enhanced survival and reduce the prostate cancer xenografts growth among athymic mouse group [133].

### 3.4. Oral Cancer

Oral cancer is the 6th common cancer around the globe [134]. In-vitro researches demonstrated the anti-proliferative effect of Kmp on oral squamous cell cancer (SCC) cells such as SCC-4, -25, -QLL1, -1483, oesophageal squamous cell carcinoma such as Eca109 cells, oral cavity tumor cells such as PCI13 and pharyngeal squamous carcinoma cells such as FaDu and inhibited cell invasion and migration, formation of clones and caused apoptosis [107,134,135,136]. Kmp triggered G0-G1 stage cell-cycle arrest and down-regulated the expression level of Bcl2, MMP2, hexokinase2 (HK2) and c-Jun and enhanced activation of EGFR, phosphorylation of ERK-1/2, glucose-uptake and up-regulated the expression of proteins PARP, caspase-9, -3 and Bax [107,134,135,136]. The anti-cancer properties of Kmp were verified in mouse xenograft models which revealed the capability of Kmp to substantially inhibit the tumor growth in combination with reduction in activity of EFGR and expression level of HK2 among cancerous tissues [135].

### 3.5. Gastric Cancer

Several studies observed the anti-proliferative activities of Kmp in SGC7901 and MKN29 stomach cancerous and promoted the G2-M stage cell cycle arrest, cell death and autophagy in these cancer cell lines [137,138]. Caused autophagic-cell death was associated with the up-regulation of PARP, IRE1-CHOP/JNK, signalling pathways, caspase-9, -3 and Bax and down-regulation expression level of Bcl2, phosphorylated-ERK, phosphorylated-AKT, CDK-1, cyclin-B1, p62, prostaglandin-endoperoxide synthase 2 [137,138].

### 3.6. Breast Cancer

Breast cancer is one of the most prevalent cancers among females in the world with significantly high mortality rate. Despite latest progress in early detection and therapeutic strategies, prevalence and mortality rate increasing continuously [139]. At concentration in micro molars (μM), Kmp efficiently prevents the breast cancerous cells growth such as MCF7, MDA-MB231 [140,141,142]. In addition, Kmp significantly prevents the bisphenol-A (and endocrine disrupting chemical) and triclosan-stimulated antiapoptotic activities [143], which initiates apoptosis, G2-M phase cell cycle arrest and DNA-fragmentation at sub G0 stage. Kmp reduces the level of antiapoptotic proteins including cyclin-A, -B, -E, -D1, CDK-1, phospho-AKT, Bcl2, Serine/threonine-protein kinase PLK1, phospho-MEK-1/2 and cathepsin-D [108,140,141,144,145,146,147] and enhances the level of proapoptotic proteins and enzymes including caspase-7, -9, -3, phospho-ATM, PARP, BAX, p53 [144,145]. It has been observed that Kmp reduced invasion and migration of cells among triple negative breast (TNB) cancerous cells in comparison with healthy cells [142]. These results described that Kmp downregulates RhoA protein and activates Rac-1 among TNB cancer cells and also activates HER-2-silence in SKBR-3 cells and ER-PR-silence in non TNB cells [142], and this indicates that the anti-proliferative effect of Kmp is initiated through estrogen receptor (ER)-dependent pathway which facilitates cell processes such as proliferation, development and differentiation [148]. Additionally, Kmp substantially triggers MAPK-cascades, and these are vital signalling pathways play an important role in regulation of differentiation, proliferation, and survival in healthy cells. Certainly, Kmp also initiates ERK along with ELK-1 and MEK-1 and reduces metastasis and EMT. After activation, MAPK signalling pathways causes activation of MMP-9 and -2, cathepsin-D and -B, AP1 which ultimately decreases invasion, adhesion, migration of cells [149,150,151,152].

### 3.7. Leukaemia

Acute promyelocytic leukaemia is a destructive disorder and characterised by defects in apoptotic pathway and growth of cells [153]. Kmp in dosage-dependent manner (12.5 to 100 μM) reduced the viability of cells among leukaemia cells such as NB-4 and HL60 [153,154]. Kmp downregulates the expression of proteins linked with phosphorylated-ATM, O^6^ methylguanine DNA methyltransferase (MGMT), p53, mediator of DNA damage checkpoint 1, phospho-ATR, DNA-dependent-protein kinase, DNA-repair mechanism, AKT, ATP Binding Cassette Subfamily C Member 1, Bcl2 genes expression and encourages G2/M stage cell cycle arrest and apoptosis. Kmp also upregulates the expression of phospho-p53, caspase-8, -3, cytochrome-c and phospho-H2AX [153,154,155,156]. The biomarker of cancer cell lines is not always directly referred to the anti-cancer event but a study observed that Kmp decreased the β-hexosaminidase release as a marker of de-granulation among leukemic cells such as RBL2H3 among mouse models [157] and enhanced the development of secretory granules in human leukaemia cells such as HMC1 [158]. A study on rat model of leukemia, found that kmp decreases the release of beta-hexosaminidase as a marker of degranulation in basophilic leukemia (RBL-2H3) cells, and increased the accumulation of mediators and the secretory granule development in human leukemic mast cells (HMC-1) [27].

### 3.8. Colon Cancer

Colon cancer is one of the most common cancers prevalent globally. The high incidences are often linked with western-style diet and intake of meat-dominant diet [159]. It has been stated that Kmp possesses cytotoxic effects on several colon cancerous cells such as HCT15, LS174T, HT29, SW40 and HCT-116 [160,161,162]. Several studies reported that Kmp in combination with 5Fluorouracil (5FU) among LS174T cells exhibited anti-proliferative effects [160]. Moreover, when Kmp combined to tumor necrosis factor-related apoptosis-inducing ligand (TRAIL) resulted in apoptosis in colorectal cancerous cells via upregulation of death-receptor 5 (DR5) and receptors of TRAIL which increased the activity of TRAIL. Kmp causes G2-M stage cell cycle arrest and apoptosis and decreases invasion and migration of cells [160,161,162]. Kmp also prevented production of ROS and regulated the expression level of PI3K-AKT, JAK-STAT3, H2AX, MAPK, p-p38, PARP, caspase-7, -9, -3, -8, Bcl2, p21, p53-upregulated-modulator-of-apoptosis, ERK1/2, NF-κB and cytochrome-c release. Kmp decreased expression of heregulin-β (HRG-β), CDK-2, cyclin-B1, -E, -A, -D1, CDK-2, -4, CDC-25C, -2 and Gap junction alpha-1 protein. Kmp also increased the cleavage of PARP and repressed the retinoblastoma protein phosphorylation [63,160,161,162,163,164,165]. In a study it was found that by modulating miR-339-5p-hnRNPA1/PTBP1-PKM2 axis, kaempferol inhibits glycolysis and colon cancer growth, which reveals a new explanation for the molecular mechanism underlying kaempferol anti-tumor [166].

### 3.9. Brain Tumor

Glioblastoma (GB) is the most destructive and common form of brain tumor which is malignant and made from of connective tissue [167,168]. Several studies observed that Kmp prevented migration and growth of GB cells and kmp-loaded mucoadhesive nano emulsion also prevented the growth of glioma tumor cells [169,170,171]. Kmp can also induces apoptosis and generation of ROS by reducing concentration of thioredoxins, activity of superoxide dismutases and by increasing the level of Bcl2, caspase-8, -3, antiapoptotic proteins such as XIAP and survivin, PARP expression, proinflammatory cytokines such as Monocyte chemoattractant protein 1 (MCP-1/CCL2), IL-8, -6, decrease in AKT and ERK signalling pathways phosphorylation and de polarization of MtMP [169,170,171,172]. 

### 3.10. Pancreatic Cancer

Pancreatic cancer is one of the major causes of cancer related deaths around the globe and have worst prognosis [173]. Kmp in dosage-dependent manner prevents the pancreatic cancerous cells growth in PANC-1, MIA PaCa-2 and SNU213-pancreatic cancer cell line by causing apoptosis [173] and efficiently preventing ERK-1/2, EGFR-related AKT and Src signalling pathways and migration of cells [174]. Kmp can improve the repressive activities of regulatory T-cells by enhancing the expression levels of forkhead box P3 (FOXP3) [175,176].

### 3.11. Bladder Cancer

Bladder cancer is the highly prominent cancer of urinary tract [177]. Kmp inhibits growth of urinary bladder cancerous cells by encouraging apoptotic pathway and cell-cycle arrest [177,178,179,180]. It has been observed that Kmp can upregulate expression level of p38, phosphorylated-BRCA1, phosphorylated-ATM, Bax, p21, DNA methylation, Bid and p53 and downregulate the PTEN-PI3K-AKT signalling pathways, cyclin-D1, B-cell lymphoma-extra-large, MCL1, DNA-methyltransferase 3 beta and CDK-4 in bladder cancer cells [177,178,179,180]. These results have been supported by experiments conducted in sub-cutaneous xenografted mice model. Kmp substantially repressed the growth of tumor, invasion, and metastasis in xenografted models in comparison with untreated healthy controls. In these xenograft mice models, Kmp also downregulated the c-MET signalling pathways and growth-related markers and triggered upregulation of markers of apoptosis [178].

### 3.12. Osteosarcoma

Osteosarcoma is a bone cancer type which begins in the cells involved in bone formation. This cancer type is highly metastatic and infects soft and bone tissues proliferate to the lungs. It occurs mostly in long body bones like leg-bones and rarely occur in soft tissues outside the bones. It commonly happens in adolescents and young adults, but it can occur in older adults too [181]. Kmp in dosage-dependent manner prevents the growth of cancerous cells in bone cancerous cell lines such as HOB, 143B, U2OS and migration of U2OS cells with poorer toxicity in human fetal osteoblast cells [182,183]. Kmp can reduce JNK, p38 and ERK mitogen-activated protein kinase (MAPK) signalling pathways by down-regulating the MMP -9, -2, urokinase-type plasminogen activator (uPA) and activator protein-1 DNA binding activity [183]. Kmp substantially reduced the cell viability and number of viable cells and decreased the size of tumor in BALB/c-nu/nu rats transplanted with U2OS cells [182].

### 3.13. Cervical Cancer

Several studies observed that the Kmp inhibited cancerous cells growth in SiHa, KB-V1 and HeLa cervical cancerous cell lines in comparison with HFF cell line and healthy cells [112,184,185,186,187]. Kmp also triggered apoptotic pathway and G2-M stage cell cycle arrest associated with up-regulation of p53 with loss of MtMP and down-regulation of PI3K-AKT, NF-κB signalling pathways, P-glycoprotein, efflux of Rhodamine 123, Bcl2, cyclin-B1 and CDK-1 [106,112,184,185,186,187].

### 3.14. Renal Cancer

Renal cell cancer signifies the very common kidney cancer [188]. Kmp substantially prevents the growth of cancerous cells and initiates apoptotic pathway in renal cancerous cell lines such as 769-P and 786-O [189,190]. Kmp uses its anti-cancer activities via inhibiting invasion and migration of cells and enhancing the focal adhesion kinase activity [188]. Kmp upregulates the expression of p21, cyclin-B1 and cleavage of PARP and encourages the EGF receptor/p38 signalling pathway activation [189,190].

### 3.15. Ovarian Cancer

Studies utilizing human ovarian cancerous cell lines such as SK-OV-3, A2780, OVCAR 3, A2780-CP70 demonstrated that Kmp can prevent angiogenesis, proliferation, and growth of cancerous cells through reducing expression of VEGF [69]. Kmp could also encourages G2-M stage cell-cycle arrest and apoptotic pathway through up-regulation of Bax, p38, CDK1/CHK2-dependent CDC25C phosphorylation, p53, death receptor-5 and -4, gadd153, p21, ERK-1/2 and bad proteins and through down-regulation of HIF-1alpha [69,70,98,105,191,192].

**Table 2:** Kaempferol (Kmp) role in cancer management through modulating cell signalling pathways.

Types of Cancer	Mechanism/Outcome of the Study	Refs.
Hepatic cancer	Kmp in a dosage-dependent manner substantially prevent proliferation liver cancerous cells such as Huh-7, SKHEP-1 and Hep.G2	[116,117]
Hepatic cancer	Additionally, 2-acetylaminofluorene and N-Nitrosodiethylamine-stimulated hepatocellular carcinoma from mice treated with combination of Kmp and luteolin prevented cancerous cells growth and caused apoptosis	[116,117]
Lung cancer	Kmp inhibited the NSCLC A549 cancerous cells, reduced formation of colonies and caused apoptosis	[125,126,127,128,129]
Lung cancer	Kmp reduced the number of metastasis and sub-cutaneous xenograft’s volume in comparison with control groups in lung-metastasis models	[129]
Prostate cancer	Kmp in dose-dependent manner prevents the proliferation of prostate cancer cells, through up-regulation of expression levels of PARP and caspase-3, -9, -8 proteins	[131,132]
Prostate cancer	Prostate cancerous cells transcriptome is significantly influenced by treatment of Kmp as it downregulated the expression of androgen-receptor genes	[133]
Oral cancer	In-vitro research demonstrated the anti-proliferative effect of Kmp on oral squamous cell cancer (SCC) cells such as SCC-4, -25, -QLL1, -1483, oesophageal squamous cell carcinoma such as Eca109 cells, oral cavity tumor cells such as PCI13 and pharyngeal squamous carcinoma cells such as FaDu and inhibited cell invasion and migration, formation of clones and caused apoptosis	[134,135,136]
Gastric cancer	Several studies observed the anti-proliferative activities of Kmp in SGC7901 and MKN29 stomach cancerous and promoted the G2-M stage cell cycle arrest, cell death and autophagy in these cancer cell lines	[137,138]
Breast cancer	At concentration in micro molars (μM), Kmp efficiently prevents the breast cancerous cells growth such as MCF7, MDA-MB231	[140,141,142]
Leukaemia	Kmp in dosage-dependent manner (12.5 to 100 μM) reduced the viability of cells among leukaemia cells such as NB-4 and HL60	[153,154]
Leukaemia	Kmp decreased the β-hexosaminidase release as a marker of de-granulation among leukemic cells such as RBL2H3 among mouse models, and enhanced the development of secretory granules in human leukaemia cells such as HMC1 (The biomarker of cancer cell lines is not always directly referred to the anti-cancer event)	[157,158]
Colon cancer	when Kmp combined to tumor necrosis factor-related apoptosis-inducing ligand (TRAIL) resulted in apoptosis in colorectal cancerous cells via upregulation of death-receptor 5 (DR5) and receptors of TRAIL which increased the activity of TRAIL	[159]
Brain tumor	Kmp prevented migration and growth of GB cells and kmp-loaded mucoadhesive nano emulsion also prevented the growth of glioma tumor cells	[169,170,171]
Pancreatic cancer	Kmp in dosage-dependent manner prevents the pancreatic cancerous cells growth in PANC-1, MIA PaCa-2, and SNU213-pancreatic cancer cell line by causing apoptosis and efficiently preventing ERK-1/2, EGFR-related AKT and Src signalling pathways and migration of cells	[173,174]
Bladder cancer	Kmp inhibits growth of urinary bladder cancerous cells by encouraging apoptotic pathway and cell-cycle arrest	[177,178,179,180]
Osteosarcoma	Kmp in dosage-dependent manner prevents the growth of cancerous cells in bone cancerous cell lines such as HOB, 143B, U2OS and migration of U2OS cells with poorer toxicity in human fetal osteoblast cells	[182,183]
Cervical cancer	Kmp inhibited cancerous cells growth in SiHa, KB-V1 and HeLa cervical cancerous cell lines in comparison with HFF cell line and healthy cells	[186,187]
Renal cancer	Kmp substantially prevents the growth of cancerous cells and initiates apoptotic pathway in renal cancerous cell lines such as 769-P and 786-O	[189,190]
Renal cancer	Kmp uses its anti-cancer activities via inhibiting invasion and migration of cells and enhancing the focal adhesion kinase activity	[188]
Ovarian cancer	Kmp can prevent angiogenesis, proliferation, and growth of ovarian cancerous cells through reducing expression of VEGF	[69]

## 4. Bioavailability of Kaempferol

The bioavailability of ingested natural materials is associated with their absorption scope and concentration [193,194]. Factors like lipophilicity, permeability, efflux, and uptake by transporters influence the amount of every compound which is taken up by mesentery and transmitted to hepatic tissue via cells of intestine [195,196,197]. Till now, lot of studies have been done describing the in-vitro effects of flavonoids such as Kmp. Although it is still debatable whether Kmp is efficient in helping actual cancerous patients. Less consumption of vegetables has been constantly connected with enhanced cancer risk. Kmp is poorly absorbed with very deprived oral bioavailability, and it is usually metabolized into glucuronide, methyl, or sulphate forms [198,199]. Kmp efflux has been reported to limit its role as an anti-cancer agent [26,199]. Numerous population-based studies have verified that Kmp rich diet decreases risk of cancer among smokers [176,200]. These results can be partly described by Kmp’s disruption of aryl-hydrocarbon receptor (AHR) signalling pathway. Human carcinogenic agents like substances found in smoke of cigarettes activated AHR signalling pathway in humans [201]. These carcinogens form complex with AHR, which translocated to nucleus and encourage carcinogenic genes expression. Kmp functions to prevent AHR and carcinogenic substance binding, thus preventing transformation of cell bring in by use of cigarettes [202]. On the other hand, uncertain outcomes have been reported by the studies concentrating on the non-smoking individuals. Several potential researches showed that in recent years, Kmp intake significantly decreased the cancer risk among American woman nurses [203]. This indicates that Kmp as low-cost, non-toxic dietary element is a promising candidate for the chemo prevention of ovarian cancer. In contrast, a few researchers have observed very restricted help for chemo prevention via flavonoid-rich diet [204], but some claim no link exists between content of flavonoid and risk of cancer [205]. Similar to all the substances, flavonoids are also administered orally, and they first pass metabolism via wall of intestine and liver [198]. Flavonoid is identified as a foreign substance by body, human cells have several pumps intended to guide these foreign substances outside of membranes and cells [206]. Kmp is inadequately absorbed in the blood and can’t make its path into the cells, where it can prevent functions of some proteins and influence signalling pathways. Due to these effluxes, anti-cancer effects of Kmp might not be felt by body [207].

But latest developments have reported hope to overcome these hurdles in bioavailability. Breast cancer-resistance protein/ATP-binding cassette super-family G member-2 (BCRP/ABCG-2) is a transporter protein which can remove host of toxic substances from cell comprising Qu, a different flavonoid which have promising future in cancer treatment. But is has been reported that Kmp has a greater affinity for BCRP/ABCG-2 as compared to Qu. Studies found that anti-cancer affinity of Kmp increased, in combination with other anti-cancer agents. For instance, Kmp and Qu combination substantially increases the anti-cancer effects of Qu via obstructing the Qu efflux which allow Qu to stay inside and influence the signalling pathways [26,189]. Hence, Kmp might probably be in combination with other components or flavonoids exhibit much greater affinity for BCRP/ABCG-2 and that would put Kmp in the cancerous cells to cause destruction. Additionally, Kmp has been observed to reduce level of mRNA of ATP Binding Cassette Subfamily C Member 6 (ABCC-6), another ATP binding cassette transport protein coding gene [108]. ABCC-6 is associated with the transport of several chemotherapeutic drugs such as cisplatin to the outside of cell [204]. It has been observed that Kmp administration considerable increase the cytotoxic efficiency of cisplatin among cancerous cells [110]. Instead of many pharmacological properties, Kmp usage in biomedical applications is less, because it has poor water solubility, poor permeability, instability of chemicals in water alkaline medium, extensive metabolic processing before entering the systemic circulation. Hence, it has been reported that Kmp can enhance the bioavailability of other components used in the cancer treatment. Recently researchers working on a new approach to tackle this issue, is the development of nanoparticles as regulated drug delivery systems for increasing the oral bioavailability of hydrophobic and lipophilic drugs such as KFP. In research it was found that encapsulation of Kmp in NPs provides a potential platform for oxidative stress induce liver injury [208]. Another study confirmed that kaempferol-coated AgNPs can induce a potential anti-cancer effect in HepG2 cells via oxidative stress-mediated apoptosis [209]. Transporters such as BCRP/ABCG-2 are a promising therapeutic research aim for enhancing the access of body to Kmp and other flavonoids [4]. 

## 5. Conclusions

Plant-derived substances have been widely studied for cancer therapy in recent time. Substances such as kaempferol have significant capabilities to prevent cancer such as anti-proliferation, anti-inflammation, cell cycle arrest and pro-oxidation and got attention as a promising cancer treatment. Various techniques and processes have been developed by researchers to study the capabilities of natural chemo preventers which improve the impact of other chemo-therapeutic treatment by reducing their toxicity and enhancing their effects. Kaempferol has demonstrated to substantially affecting several cancer-related mechanisms, pathways and exhibited inhibitory effect on various cancer types including breast, hepatic, colon, lung, prostate, bladder, ovarian, oral, gastric, renal cancers. The review thus presents cumulative compendium of extensive research investigating the potential therapeutic role of kmp, in treatment of various types of cancers. Kaempferol also linked with some limitations mainly related to limited research in various domains of cancer, poor-absorption, and poor bioavailability. Hence, based on various therapeutic benefits of kmp, it strongly supports the development of clinical trials, incorporation of new approaches like nano technology to its application which can significantly enhance the potential of quercetin as powerful therapeutic agent. It can open new horizons in effective utilization, wider applicability, and better bioavailability of kmp as a potent natural chemo preventer alone or in from of combination drug for better prevention and management of cancer. 

## Data Availability

Not applicable.
